# Circulating Chromogranin A as a Surveillance Biomarker in Patients with Carcinoids—The CASPAR Study

**DOI:** 10.1158/1078-0432.CCR-24-1875

**Published:** 2024-10-25

**Authors:** Qing H. Meng, Thorvardur R. Halfdanarson, Joshua A. Bornhorst, Henning Jann, Shagufta Shaheen, Run Zhang Shi, Andrej Schwabe, Katrin Stade, Daniel M. Halperin

**Affiliations:** 1 Department of Laboratory Medicine, The University of Texas MD Anderson Cancer Center, Houston, Texas.; 2 Division of Medical Oncology, Department of Oncology, Mayo Clinic, Rochester, Minnesota.; 3 Department of Laboratory Medicine and Pathology, Mayo Clinic, Rochester, Minnesota.; 4 Division of Hepatology and Gastroenterology, Medical Department, Charité-Universitätsmedizin, Berlin, Germany.; 5 Division of Oncology, Department of Medicine, Stanford University School of Medicine, Stanford, California.; 6 Department of Pathology, Stanford University School of Medicine, Stanford, California.; 7 B·R·A·H·M·S GmbH, part of Thermo Fisher Scientific, Hennigsdorf, Germany.; 8 Department of Gastrointestinal Medical Oncology, The University of Texas MD Anderson Cancer Center, Houston, Texas.

## Abstract

**Purpose:**

Gastroenteropancreatic neuroendocrine tumors (GEP-NET) are relatively indolent but can be more aggressive. The current recommendations for using serum chromogranin A (CgA) for patients with GEP-NET are equivocal. This study was designed to validate an automated CgA immunofluorescence assay for monitoring disease progression in patients with GEP-NET.

**Patients and Methods:**

A prospective, multicenter, blinded observational study was designed to validate an automated CgA immunofluorescence assay for monitoring disease progression in patients with GEP-NET. Tumor progression was evaluated with RECIST 1.1 by CT/MRI. An increase ≥50% above the prior CgA concentration to a value >100 ng/mL in the following CgA concentration was considered positive.

**Results:**

A total of 153 patients with GEP-NET were enrolled. Using the prespecified cut-off of CgA change for tumor progression, specificity was 93.4% (95% confidence interval, 90.4%–95.5%; *P* < 0.001), sensitivity 34.4% (25.6%–44.3%), positive predictive value 57.9% (45.0–69.8), negative predictive value 84.3% (80.5–87.6), and AUC 0.73 (0.67–0.79).

**Conclusions:**

Changes in serial measurements of serum CgA had a favorable specificity and negative predictive value, making this test a useful adjunct to routine radiographic monitoring.

**Translational Relevance**

Clinical utility of chromogranin A (CgA) measurements in patients with gastroenteropancreatic neuroendocrine tumors (GEP-NET) has long been a controversial issue. In this study, we wanted to explore whether changes in serum CgA biomarker concentrations detect disease progression in patients with GEP-NET during follow-up. In this prospective, observational validation study that included 153 adults, CgA concentration changes above a prespecified cut-off had a diagnostic specificity of 93.4% and a negative predictive value of 84.3% for tumor progression. In patients with GEP-NET, changes in the measurement of serum CgA as an adjunct to imaging may improve clinical decision-making.

## Introduction

Neuroendocrine neoplasms, including gastroenteropancreatic neuroendocrine tumors (GEP-NET) constitute approximately 2% of all neoplasms (1). However, the incidence is on the rise (2–6). Epidemiologic and prospective data suggest that the rate of tumor progression is highly variable among patients (7). Currently, a GEP-NET diagnosis relies on patient demographics, symptom assessment, imaging results by CT or MRI, and laboratory testing (8–10). Patients with advanced disease are regularly monitored through repeated radiologic imaging, typically every 3 to 12 months. This wide interval reflects the disease heterogeneity and lack of interval consensus based on clinical variables alone. Biochemical testing during follow-up may include monitoring the concentration of circulating chromogranin A (CgA), a 439 amino acid glycopeptide from the granin family. CgA is a well-known biomarker for GEP-NET with a reported diagnostic sensitivity and specificity between 60% and 90% (11, 12). CgA serves as a tissue biomarker but may also be measured as a circulating peptide in serum or plasma.

Clinical evidence currently available on the potential diagnostic or prognostic value of serum CgA is inconsistent because some reports support the use of CgA in GEP-NET evaluation (12–14), whereas others do not (15–17). This may be because most of the studies are retrospective, with small sample sizes and heterogenous study populations. Additionally, incomplete data reporting and noncompliance with standards for study quality, such as the Standards for Reporting Diagnostic Accuracy Studies criteria (18), represent further hurdles to study data evaluation. As a result, CgA’s clinical utility is variably represented in current medical guidelines for the management of patients with GEP-NET. Whereas the European Neuroendocrine Tumor Society (refs. 11, 19) and European Society for Medical Oncology (ref. 9) guidelines support serial measurements of circulating CgA for the management of patients with GEP-NET, the National Comprehensive Cancer Network (ref. 10) and North American Neuroendocrine Society (ref. 8) guidelines do not contain a clinical algorithm for CgA. Therefore, the value of serum CgA measurements in the surveillance and monitoring of GEP-NET is not widely endorsed. The present study aimed to validate an automated CgA immunoassay to monitor disease course in patients with primary, well-differentiated GEP-NET (grade 1 and grade 2). The study success criteria were based on the diagnostic performance of CgA as estimated in a retrospective pilot study. This is the first prospective, multicenter validation of a clinical algorithm with a prespecified cut-off for the interpretation of the change in CgA levels in patients with advanced GEP-NET.

## Patients and Methods

### Study design and participants

The CASPAR study was a prospective, multicenter, observational study of 153 adult patients with GEP-NET conducted from January 2019 to December 2021 at three study sites in the United States (The University of Texas MD Anderson Cancer Center, Mayo Clinic, and Stanford University Medical Center) and one study site in Germany (Charité-Universitätsmedizin Berlin). Patients aged 18 years or older with a diagnosis of advanced primary well-differentiated grade 1 or grade 2 GEP-NET located in the jejunum, ileum, colon, rectum, duodenum, appendix, stomach, or pancreas and a willingness to participate in this study were consecutively enrolled. Patients with no measurable disease based on RECIST 1.1 criteria as well as those with other active malignancies, severe renal or liver dysfunction, severe gastrointestinal or cardiovascular disease, pregnancy, or any signs of chronic alcohol or substance abuse were excluded. Patients who wished to participate in the study but were receiving active treatment with proton pump inhibitors (PPI), corticoids, or H_2_-receptor antagonists were asked to stop intake 3 weeks before their blood samples were collected. The study was registered on ClinicalTrials.gov (NCT03817866), approved by the Ethics Committees of all participating sites, and performed in accordance with the Declaration of Helsinki, Good Clinical Practice guidelines, and all local regulations. Written informed consent was obtained from each patient after a full explanation of the purpose and nature of all the procedures to be used and before the performance of any study procedure.

### Study procedures

At the time of study inclusion, patient demographics, clinical assessment, initial disease status, presence of an autonomous hormone secretion syndrome, laboratory values, tumor therapies, concomitant medications, and baseline RECIST 1.1 measurements via CT or MRI were recorded. At each subsequent follow-up visit (usually 3–6 months apart), disease progression status, laboratory values, tumor therapies (including therapy decisions), and concomitant medications were recorded, and CT/MRI images were obtained and evaluated according to RECIST 1.1 criteria. Overall survival status was recorded as of the final visit.

### Laboratory analyses

During each visit, 8.5 to 10 mL of whole blood was drawn, and samples were processed according to the sites’ standard operating procedures to obtain serum. Routine clinical laboratory parameters were measured. CgA concentrations were measured with the automated B·R·A·H·M·S CgA II KRYPTOR immunofluorescence assay on a B·R·A·H·M·S KRYPTOR Compact Plus instrument. Samples were analyzed either fresh or after storage at −80°C typically within 3 weeks of collection. The CgA assay results were not part of the patient assessment, and radiologists performing the RECIST 1.1 assessments were blinded to CgA values.

### Outcomes

The primary outcome was binary disease status according to the RECIST 1.1 criteria (progression and no progression). In brief, patients were categorized into groups. Group 1 had no evidence of disease (complete response, as per RECIST 1.1 criteria), group 2 had “evidence of residual but stable disease” (stable disease and “non–complete response/non–progressive disease”), group 3 had “evidence of progressive disease” (progressive disease), and group 4 had “responsive disease” (partial response). Groups 1, 2 and 4 were classified as “no progression” and group 3 as “progression.”

### Statistical analyses

Relative change of CgA concentration between consecutive visits was the primary predictor. The following definitions of relative change were used: (i) “binary ΔCgA” was defined as positive if the CgA concentration increased from previous to current visit by more than the prespecified cut-off of 50% to an absolute value greater than the threshold (i.e., 100 ng/mL). The cut-off and CgA threshold were derived from the pilot study. (ii) “Simple ΔCgA” was computed from the concentrations of previous and current visits [(CgA)_previous, (CgA)_current] as [(CgA)_current − (CgA)_previous]/(CgA)_previous × 100%. (iii) For “continuous ΔCgA,” (CgA)_previous was replaced in the formula by 100 ng/mL if (CgA)_previous was less than 100 ng/mL. This CgA threshold of 100 ng/mL was introduced to avoid artificially large ΔCgA values in case of low CgA concentrations. For regression analysis, log-transformed CgA ratio was used as a predictor instead of continuous ΔCgA, with CgA ratio determined by converting continuous ΔCgA using ΔCgA/100% + 1.

The intention-to-diagnose population included all evaluable patients defined as those with at least a baseline and one follow-up visit that included both RECIST assessments and CgA measurements. Primary analysis was focused on the endpoint tumor progression and comprised (i) testing the association with binary ΔCgA by Pearson’s χ^2^ test, (ii) computing diagnostic performance measures, (iii) univariate logistic regression analysis, and (iv) receiver operating characteristics (ROC) analysis. Additional analyses included Kaplan–Meier plots, Cox proportional hazards regression, and log-rank tests for the endpoint progression-free survival for baseline covariates and ΔCgA. Continuous variables were reported as median values with the IQR (25th–75th percentile). Differences between patient groups were analyzed using the Mann–Whitney U test. Statistical analyses were conducted using R software (version 4.0.5) and R packages Hmisc (version 4.5-0), pROC (version 1.17.0.1), ggplot2 (version 3.3.5, RRID: SCR_014601), and survminer (version 0.4.9; ref. 20).

Study success was prespecified as (i) significant association between tumor progression and binary ΔCgA, (ii) a lower limit of the 95% confidence interval (CI) of specificity greater than 87.5%, and (iii) a lower limit of the 95% CI of sensitivity greater than 15.5%. Statistical testing was two-sided (if applicable), and *P* values below 5% were considered significant. Please see Supplementary Materials for details of the statistical approach, sample size calculations, and pilot study.

### Data availability

Data collected for the study, including participant data with identifiers, are not publicly available to respect patients’ rights to confidentiality and privacy. Deidentified patient data, the study protocol, statistical analysis plan, and informed consent forms can be requested by email to CASPAR.study@thermofisher.com and will be made available after an approval of a proposal with a signed data access agreement. Data will be available upon publication of the article and for 6 years after.

## Results

### Inclusion and patient demographics

**Figure 1.**
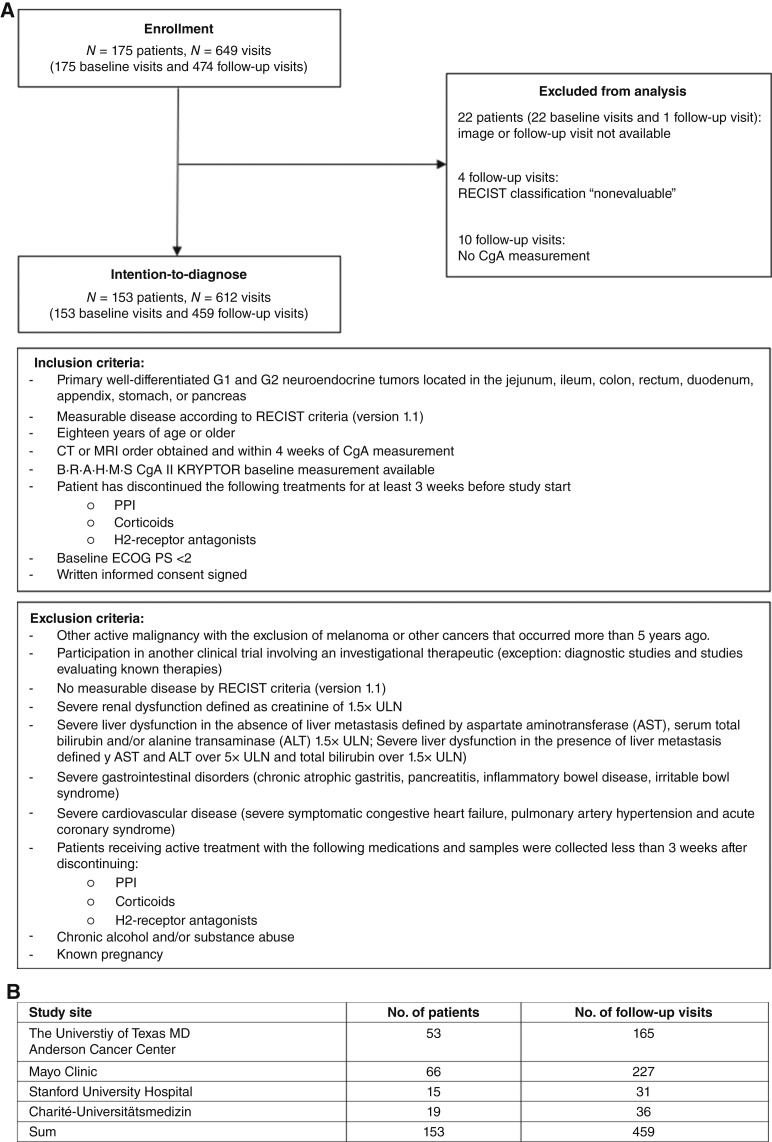
**A,** Clinical trial profile. Inclusion and exclusion criteria. **B,** Study site visit information for intention-to-diagnose population. ECOG, Eastern Cooperative Oncology Group.

From January 2019 to December 2021, 175 patients were assessed for study eligibility and were subsequently enrolled (Fig. 1A). Of these, 22 patients with 23 corresponding visits were completely excluded from analysis due to unavailable baseline CT/MRI images or unavailable follow-up visits. Four follow-up visits were excluded due to RECIST 1.1 classification “nonevaluable,” and 10 follow-up visits due to unavailable CgA measurements. One patient was lost to follow-up, and 13 patients died during the study (data were excluded). The number of enrolled patients and follow-up visits varied across the four study sites (Fig. 1B). The median time from the initial diagnosis to the enrollment day was 38.7 months (1,174 days, IQR 423–2,683 days). Clinical characteristics at baseline of the remaining 153 patients, the intention-to-diagnose population, are presented in Table 1. The median age was 63 years (IQR, 54–69 years), and 59% were male. Most patients were classified as White (94%). Patients were in the study for up to 32 months (median, 11 months). Sixty-seven percent of the patients had an Eastern Cooperative Oncology Group performance score of 0 and 33% a score of 1. The median Ki-67 index was 5% (IQR, 2%–9%), with 40% having grade 1 (G1) disease and 60% having grade 2 (G2) disease. The primary tumor site was identified either in the small intestine (61%), pancreas (36%), large intestine (2%), or stomach (1%). The primary tumor stage was classified as stage IV in 91% of patients. Approximately two thirds (63%) had nonfunctional tumors. Eighty-eight percent had liver metastasis, and 41% had lymph node metastasis. The overall number of progressions per patient ranged from one (14%) to more than two (8%), more than three (3%), and more than three (5%); and 70% did not have any tumor progression (data not shown). The application of concomitant medication ranged from 98% to 100% across sites. Eighty-one percent received drug therapy, such as treatment with somatostatin analogue (SSA, 73%), and chemotherapeutic agents were applied in 26% of the patients. Radiation treatment (including peptide receptor radionuclide therapy) was performed in 7%.

**Table 1.** Patient characteristics at baseline.

Characteristic	Value or number of patients (*n* = 153)	%
Age (years)		
Median	63	NA
IQR	54–69	NA
Sex		
Male	91	59
Female	62	41
Ethnicity^a^		
White	143	94
Other	5	3
Black or African American	4	3
Asian	1	1
ECOG performance status		
0	103	67
1	50	33
Primary site of tumor		
Pancreas	55	36
Small intestine (duodenum, jejunum, and ileum)	94	61
Large intestine (appendix, cecum, and colon)	3	2
Stomach	1	1
Metastatic sites^b^		
Liver	134	88
Lymph node	62	41
Other	46	30
None	7	5
Lung	2	1
Ki67 index		
Median	5	NA
IQR	2–9	NA
Tumor grade		
G1	61	40
G2	92	60
TNM stage		
0–III	14	9
IV	139	91
Autonomous hormone secretion syndrome		
Nonfunctional tumor	96	63
Functional tumor	57	37
Method of assessment		
CT	86	56
MRI	67	44
Concomitant medication		
Yes	151	99
No	2	1
Any tumor therapy		
Yes	124	81
No	29	19
Type of tumor therapy^b^		
Somatostatin analogue	112	73
Chemotherapy	40	26
Radiation therapy, including PRRT	11	7
Any procedure		
Yes	41	27
No	112	73
Type of procedure		
Surgery	40	26
Local therapy	1	1
Baseline CgA (ng/mL)		
Median	125.8	NA
IQR	63.8–279.5	NA

Abbreviations: ECOG, Eastern Cooperative Oncology Group; NA, not applicable; PRRT, peptide receptor radionuclide therapy; TNM, tumor–node–metastasis.

a More than 100% due to rounding.

b Selections of more than one category allowed.

### Diagnostic performance of the CgA test

A binary ∆CgA test result (see “Patients and Methods”) was compared with a patient’s disease state according to RECIST 1.1 criteria (progression vs. no progression) determined from the same follow-up visit. As shown in Table 2, for a total of 459 follow-up visits, 96 (21%) visits with tumor progression and 57 (12%) visits with positive ∆CgA were observed. A total of 339 ∆CgA tests were true negatives (i.e., no progression and test-negative ΔCgA), 63 false-negatives (progression but test-negative), 33 true positives (progression and test-positive), and 24 false-positives (no progression but test-positive). The binary ∆CgA test results were significantly associated with tumor progression status (*P* value < 0.001), and the following performance characteristics were obtained: specificity 93.4% (95% CI, 90.4%–95.5%); sensitivity 34.4% (25.6%–44.3%); positive predictive value 57.9% (45.0%–69.8%); negative predictive value (NPV) 84.3% (80.5%–87.6%); positive likelihood ratio 5.20 (3.23–8.36); and negative likelihood ratio 0.70 (0.61–0.81). The OR for the endpoint disease state (progression or no progression) was 7.40 (95% CI, 4.12–13.49; *P* < 0.001) for test-positive versus test-negative test results and 3.09 (95% CI, 2.22–4.46; *P* < 0.001) for a doubling of CgA ratio.

**Table 2.** Contingency table and diagnostic performance metrics of intention-to-diagnose population.

	Disease status according to RECIST 1.1			
ΔCgA test results	No progression	Progression	Row sum	
Negative	339 (TN)	63 (FN)	402	
Positive	24 (FP)	33 (TP)	57	
Column sum	363	96	459	

Diagnostic performance metrics for disease status according to RECIST 1.1	Estimate (95% CI)	
Sensitivity, %	34.4 (25.6–44.3)	
Specificity, %	93.4 (90.4–95.5)	
PPV, %	57.9 (45.0–69.8)	
NPV, %	84.3 (80.5–87.6)	
LR^+^	5.20 (3.23–8.36)	
LR^−^	0.70 (0.61–0.81)	
AUC	0.731 (0.669–0.793)	
OR, test-positive vs. test-negative	7.40 (4.12–13.49)	
OR, doubling of CgA ratio	3.09 (2.22–4.46)	

Abbreviations: FN, false negative; FP, false positive; LR +/−, positive and negative likelihood ratios; PPV, positive predictive value; TN, true negative; TP, true positive.

**Figure 2.**
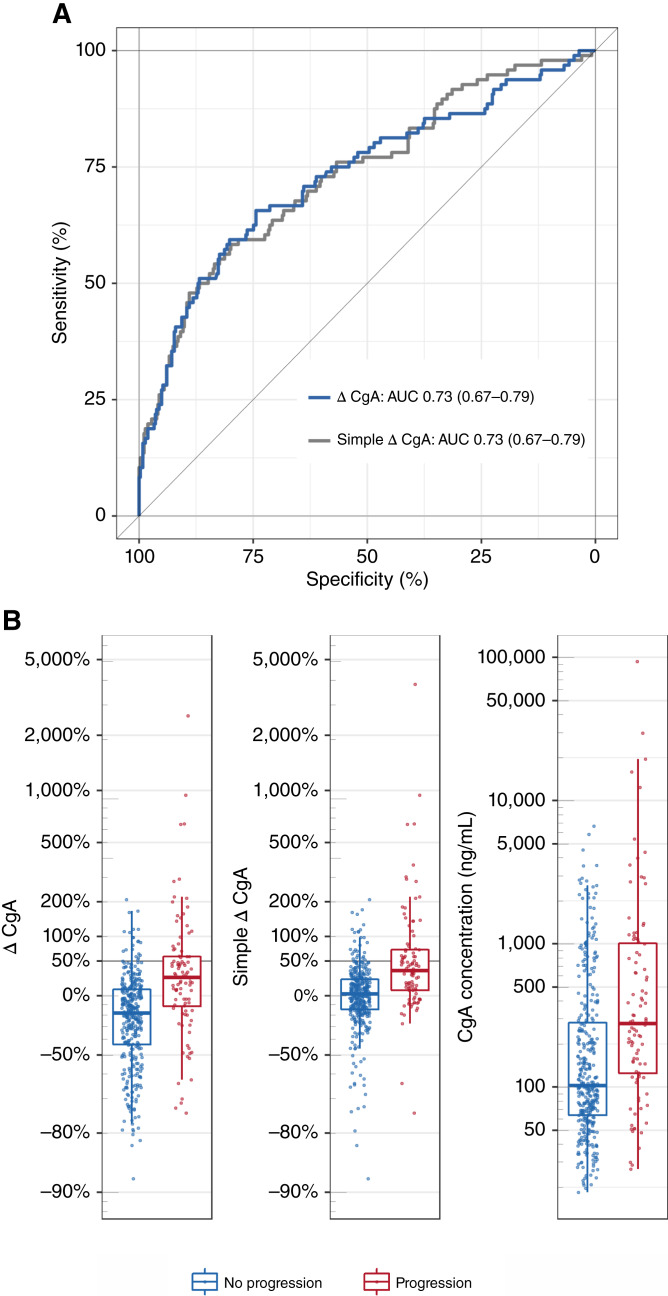
**A,** ROC curves for diagnosis of tumor progression in the intention-to-diagnose population with corresponding AUC. **B,** Box plots of continuous ΔCgA, simple ΔCgA, and CgA concentrations are shown with log-scaled axes. Strata of follow-up visits of the intention-to-diagnose population: no progression (blue) and progression (red). The horizontal gray lines show the prespecified cut-off 50% for ΔCgA.

The ability of continuous ΔCgA to discriminate between progression and no progression was further determined by ROC analysis yielding an AUC of 0.73 (95% CI, 0.67–0.79; see the blue line in Fig. 2A). A similar AUC was obtained for the simple ΔCgA, see the gray line, indicating comparability of these two methods to define CgA change (see comparison of diagnostic performance metrics in Supplementary Table S2).

Figure 2B shows box plots of continuous ΔCgA for follow-up visits with (red) and without (blue) progression. ∆CgA [Fig. 2B (left)] was on average higher for progressions with a median of 24% (IQR: −12% to 58%) compared with no progressions with a median of −18% (IQR, −44% to 8%; *P* < 0.001). A comparable distribution was obtained for simple ΔCgA [Fig. 2B (middle)]. Furthermore, CgA concentrations [Fig. 2B (right)] were on average higher by a factor of about 2.7 for progressions (median, 279 ng/mL, IQR: 125–1,012 ng/mL) compared with no progressions (median, 102 ng/mL, IQR, 64–282 ng/mL; *P* < 0.001). The CgA level was associated with the tumor burden (i.e., the sum of the length of the lesions) and increased by 13% per cm (95% CI, 11%–15% per cm, *P* < 0.001; Supplementary Fig. S1). The relationship was similar for visits with and without progression, with the CgA concentrations higher on average for visits with progression.

**Figure 3.**
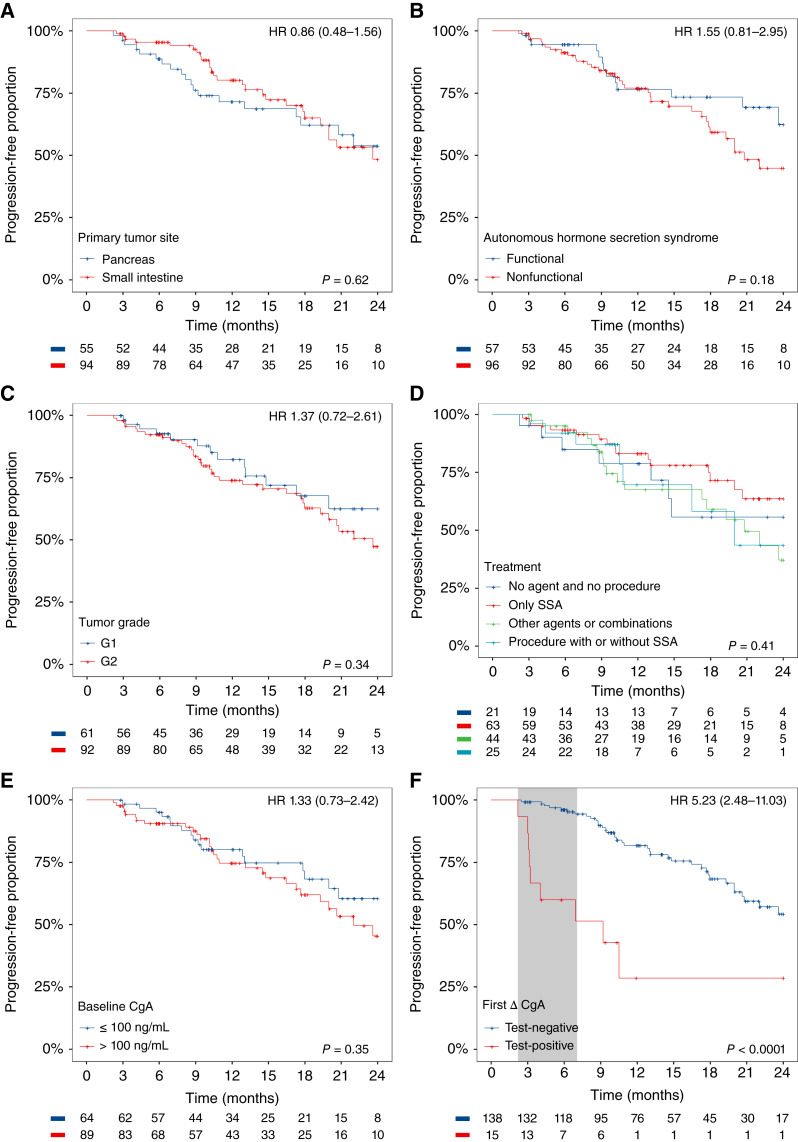
Kaplan–Meier plots with proportion of progression-free patients with GEP-NET (%) during 24 months of follow-up in the intention-to-diagnose population, with stratification by primary tumor site (**A**; largest subgroups—pancreas and small intestine), presence of autonomous hormone secretion syndrome (**B**), tumor grade (**C**), treatment type (**D**), binary CgA at baseline (**E**), and binary ΔCgA at the first follow-up visit (**F**). The gray part in **F** indicates the period when 75% of the first follow-up visits after baseline occurred in the test-positive group (2–7 months, comparable time window in the test-negative group). Below each panel, the numbers of patients at risk are indicated for each timepoint and stratum. HR for comparison of the second (red) vs. the first stratum (blue), with 95% CI in brackets; *P* values are obtained from log-rank tests. **D,** HR for comparison with stratum “no agent and no procedure:” “only SSA” 0.66 (*P* = 0.37), “other agents or combinations” 1.19 (*P* = 0.70), and “procedure with or without SSA” 1.03 (*P* = 0.96). SSA, somatostatin analogues.

Figure 3 shows Kaplan–Meier plots for the progression-free survival of patients with GEP-NET during 24 months of follow-up, stratified by the primary tumor site (A), presence of autonomous hormone secretion syndrome (B), tumor grade (C), treatment type (D), and baseline CgA (E). The time to first progression was slightly shorter for most time intervals for patients with nonfunctional tumors (vs. functional, B), grade 2 tumors (vs. grade 1, C), and baseline CgA concentrations greater than 100 ng/mL (E). (F) shows stratification by binary ΔCgA at the first follow-up visit. The curve for the test-positive group was mainly influenced by the early occurrence of progressions in this group. Eight of nine first progressions had occurred by the time of the first follow-up visit, resulting in a considerable drop of the proportion of progression-free patients to 55% at 7 months (75% of the first follow-up visits occurred between 2 and 7 months in the test-positive group, gray shaded area, and this timing was comparable with that in the test-negative group). In the test-negative group, time to progression was substantially larger, reflecting the observed performance of ∆CgA in detecting tumor progression (HR, 5.23, 95% CI, 2.48–11.03; *P* < 0.001). Additional Kaplan–Meier plots for primary tumor stage, Eastern Cooperative Oncology Group status, and Ki-67 proliferative index are shown in Supplementary Fig. S2.

## Discussion

The current recommendations for the use of serum CgA during the management of patients with GEP-NET are equivocal. This study was designed to provide critical prospective evaluation of CgA’s performance using defined criteria applied to a defined population of patients with advanced disease. Whereas one review concluded CgA as a prognostic and/or diagnostic serum biomarker that was not of value (17), another systematic review identified eight mainly retrospective studies that supported the use of CgA to detect progressive disease during patient follow-up with an overall diagnostic accuracy of 84% (16). Additional evidence from studies with mainly retrospective designs, small study populations, and often poor compliance with quality criteria for diagnostic studies may have further contributed to this issue of inconsistency. Unfortunately, this discrepancy is also reflected in current medical guidelines in Europe and the United States (8–10).

To definitively address the question whether CgA is a useful serum biomarker for GEP-NET, we initiated the first international, multicenter, prospective CgA validation study. The planning, conduct, and reporting of the CASPAR study was done according to Standards for Reporting Diagnostic Accuracy Studies criteria, including detailed study protocol, prespecified analysis, and study success criteria (18, 21). CgA results were compared with the currently best available oncologic imaging standard, RECIST version 1.1, to assess patients with GEP-NET (22).

**Figure 4.**
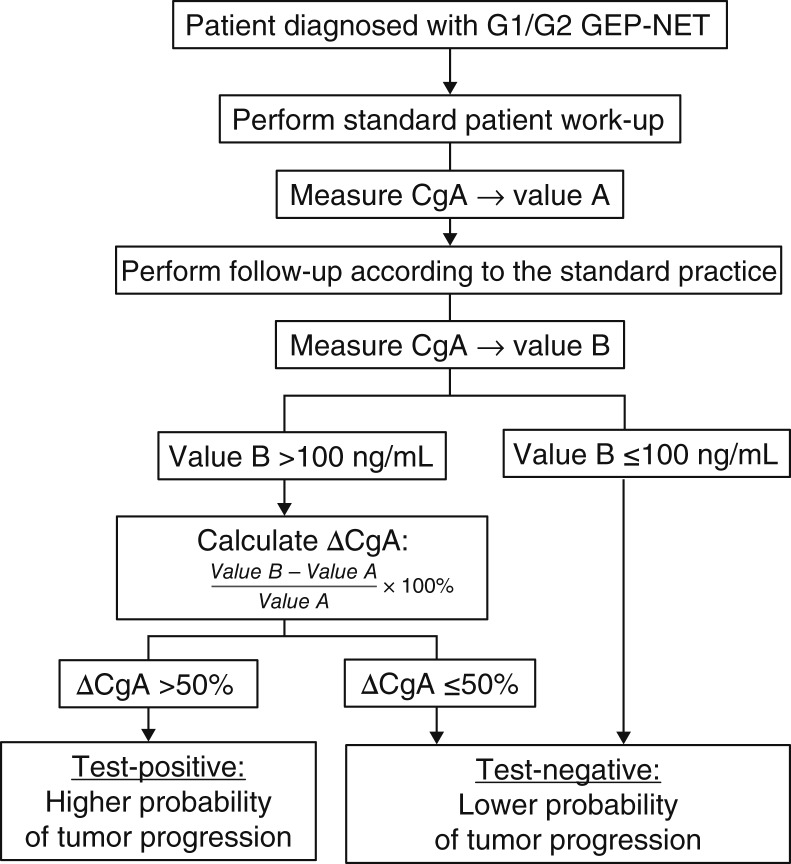
Clinical algorithm.

Our results show that CgA measurements and changes in CgA concentrations are useful during the follow-up of patients with GEP-NET. The performance of a clinical algorithm that included serial CgA measurements during patient follow-up and the prespecified cut-off of 50% was successfully validated. We concluded that defined ΔCgA and tumor progression status according to RECIST 1.1 were substantially associated and that the diagnostic performance of binary ΔCgA to diagnose progression is useful for clinical application. With the observed performance results (e.g., 84% NPV and 93% specificity), ΔCgA provides additional useful information about a patient’s disease state for clinical decision-making, for example, on imaging frequency. Furthermore, the CgA concentration was associated with tumor burden (sum of the length of lesions). In a sensitivity analysis, performance results were similar for small changes in the definition of ΔCgA (similar results for using a different or no CgA threshold at all; Supplementary Table S2). Based on these robust findings, we propose that a baseline CgA value should be considered in all newly diagnosed patients with GEP-NET. Serial serum CgA measurements could be performed at regular follow-up visits to monitor the disease course. Figure 4 introduces this novel clinical algorithm, which in combination with imaging modalities may aid in the assessment of patients’ disease progression and timing of follow-up within the range of current practice guidelines, particularly when RECIST 1.1 is not the standard of care. For a patient without known confounding comorbidities or medication exposure, and in whom the clinician has a strong prior suspicion of progressive disease, a positive ∆CgA may indicate that a progression has occurred and therefore further assessment may be warranted, including additional imaging modalities such as PET-CT if standard images were negative or of poor resolution. A negative ΔCgA, together with routine clinical assessment, may support the exclusion of tumor progression and may support a decision to perform imaging at the next suitable follow-up visit. However, serum CgA measurements are not recommended to extend imaging intervals beyond the 12-month maximum suggested in current guidelines. Symptom assessment, along with imaging, remains the primary method of clinical evaluation for these patients. Although the automated B·R·A·H·M·S CgA II KRYPTOR immunofluorescence assay used in our study is more cost-effective than manual or semiautomated CgA methods, appropriate serial testing is important to avoid both unnecessary patient anxiety and health care costs. When used adequately, such testing can potentially reduce the costs of unnecessary imaging within the suggested time intervals, resulting in significant net savings. In an independent study, Dam and colleagues published findings similar to ours that looked at disease progression at future follow-up visits for ∆CgA >25% (23). Their calculated NPV of 85% suggests a potential role for the use of negative ∆CgA in the decision of prolonging radiographic evaluation within a 12-month follow-up period.

CgA might be elevated after the use of concomitant medications such as PPI/H_2_-receptor antagonists or when other oncologic conditions are present (24). Although admission to CASPAR was possible for patients using PPI only if they terminated this medication, we cannot fully exclude the possibility that some patients resumed their medication during the study without notifying study personnel. This could be an explanation for some of the *n* = 24 false-positive ΔCgA results from our study. However, when interpreted together with symptoms and imaging data, detection of an erroneously high CgA value is highly likely. False-negative ΔCgA results (*n* = 63) are more of a concern because an asymptomatic patient with a developing disease progression could have their imaging interval prolonged, albeit still within the recommended range. Lower CgA secretion was previously observed in patients with nonfunctional NET or GEP-NET without liver metastasis (25, 26). It remains unclear if these groups contribute to the falsely low CgA values observed in our study. Nonetheless, thorough data interpretation at each follow-up visit, including symptom assessment, serum CgA concentrations, and imaging results, allows for a reliable assessment of the true disease status in patients with GEP-NET.

We used RECIST 1.1 imaging criteria to evaluate a patient’s tumor burden (22). The utility of RECIST 1.1. for use in GEP-NET has been under debate due to their heterogeneity in structure and growth kinetics (27). However, until better evaluation criteria are developed, RECIST 1.1 remains the reference standard for evaluation of this tumor type in clinical trials (28).

In conclusion, serial serum CgA measurements can aid in monitoring disease progression in patients with GEP-NET in conjunction with other clinical approaches but should not replace cross-sectional imaging. CgA can potentially guide the frequency of imaging in appropriately selected, asymptomatic patients within standard intervals.

## Supplementary Material

Supplementary Data 1 Supplementary data for CASPAR pilot study, tables and figures
