# Supplementary material for: Circulating Chromogranin A as a Surveillance Biomarker in Patients with Carcinoids—The CASPAR Study
Source: Clin Cancer Res. 2024 Oct 25;30(24):5559–67. doi: 10.1158/1078-0432.CCR-24-1875 (PMC11647202; doi:10.1158/1078-0432.CCR-24-1875)
Supplement: Supplementary Data 1 — Supplementary data for CASPAR pilot study, tables and figures [file ccr-24-1875_supplementary_data_1_suppds1.docx]

**Supplementary data**

**Details of the CASPAR pilot study**

The pilot study was a retrospective study that included 102 eligible patients with GEP-NETs of two study sites with 340 evaluable follow-up visits. Inclusion and exclusion criteria were the same as for the CASPAR study. Median age was 78 years, 47% patients were male, and median time between visits was 6 months. 62 RECIST 1.1 tumor progressions were observed, distributed over 42 patients with a maximum of three progressions per patient. Patient follow-up was up to 48 months (median 20 months). The resulting contingency table for binary ΔCgA vs tumor progression and diagnostic performance measures are shown in table S1.

**Details of the statistical approach and sample size calculations**

Primary analysis was focused on the binary endpoint tumor progression and comprised following methods. The association with binary ΔCgA was determined using Pearson’s chi-square test for count data. The diagnostic performance measures sensitivity, specificity, positive and negative predictive value, positive and negative likelihood ratio were calculated according to conventional formulas. Their CIs are reported according to the pre-specified Wilson score method^23^ which yielded similar CIs as a bootstrap re-sampling method accounting for the hierarchical structure of follow-up visits nested within patients. Univariate logistic regression models were generated to estimate odds ratios for a) binary ΔCgA and b) log transformed CgA ratio. The area-under-the-curve (AUC) value of continuous ΔCgA was obtained from Receiver Operating Characteristics (ROC) analysis. In addition to the pre-specified analysis, Kaplan-Meier plots were generated for the endpoint progression-free survival (time to first progression or death) and several categorial baseline covariates and binary ΔCgA at the first follow-up visit. For each studied variable, the corresponding univariate Cox proportional hazards regression model was generated to estimate the hazard ratio (HR) and statistical significance was determined by a log-rank test. The association between CgA concentration and tumor burden (the sum of lesion’s longest diameters per RECIST 1.1) was analyzed using an ordinary linear regression (CgA concentration log transformed).

The study sample size was determined such that estimates of specificity and sensitivity are with specified confidence above lower limit values derived from the retrospective pilot study.^22^ Seventy tumor progressions and 314 no-progressions according to RECIST 1.1 criteria were required to achieve 80% statistical power for study success based on the effect size and prevalence of progressions in the pilot study.

|  | Disease status according to RECIST 1.1 | |  |
| --- | --- | --- | --- |
| ΔCgA  test results | No Progression | Progression | Row Sum |
| Negative | 260 (TN) | 42 (FN) | 302 |
| Positive | 18 (FP) | 20 (TP) | 38 |
| Column Sum | 278 | 62 | 340 |
|  | | | |
| Diagnostic performance metrics  for disease status according to RECIST 1.1 | | Estimate (95% CI) | |
| Sensitivity, % | | 32·3 (22·0 - 44·6) | |
| Specificity, % | | 93·5 (90·0 - 95·9) | |
| PPV, % | | 52·6 (37·3 - 67·5) | |
| NPV, % | | 86·1 (81·7 - 89·5) | |
| CI, confidence interval; FN, false negative; FP, false positive; TN, true negative; TP, true positive; NPV, negative predictive value; PPV, positive predictive value. | | | |
| **Table S1_ Contingency table of CASPAR pilot study** | | | |

|  | **Pearson’s chi square Test** | **Diagnostic Performance Metrics**  estimate (95% CI) | | | | | | **ROC analysis**,  estimate  (95% CI) | **Odds ratios** | |
| --- | --- | --- | --- | --- | --- | --- | --- | --- | --- | --- |
| **CgA threshold (ng/mL)** | **P-value** | **Specificity** | **Sensitivity** | **PPV** | **NPV** | **LR+** | **LR-** | **AUC** | **test positive vs. test negative** | **doubling of CgA ratio** |
| none | <0·001 | 92·6% (89·4–94·8) | 36·5%  (27·5–46·4) | 56·5%  (44·1–68·1) | 84·6%  (80·8–87·8) | 4·90  (3·13–7·68) | 0·69  (0·59–0·80) | 0·734  (0·675–0·794) | 7·14  (4·05–12·74) | 5·00  (3·14–8·35) |
| 100·0 | <0·001 | 93·4%  (90·4–95·5) | 34·4%  (25·6–44·3) | 57·9%  (45·0–69·8) | 84·3%  (80·5–87·6) | 5·20  (3·23–8·36) | 0·70  (0·61–0·81) | 0·731  (0·669–0·793) | 7·40  (4·12–13·49) | 3·09  (2·22–4·46) |
| 185·0 | <0·001 | 94·2 % (91·3–96·2) | 27·1%  (19·2–36·7) | 55·3%  (41·2–68·6) | 83·0%  (79·1–86·3) | 4·68  (2·76–7·95) | 0·77  (0·68–0·88) | 0·720  (0·658–0·781) | 6·05  (3·23–11·46) | 2·32  (1·81–3·04) |
| PPV, positive predictive value; NPV, negative predictive value; LR +/-, positive and negative likelihood ratios; ROC, receiver-operating curve; AUC, area under the curve; CI, confidence interval | | | | | | | | | | |
| **Table S2_Comparison of diagnostic performance metrics for disease status according to RECIST 1.1 in the intention-to-diagnose population for different CgA threshold values** | | | | | | | | | | |

**Figure S1**


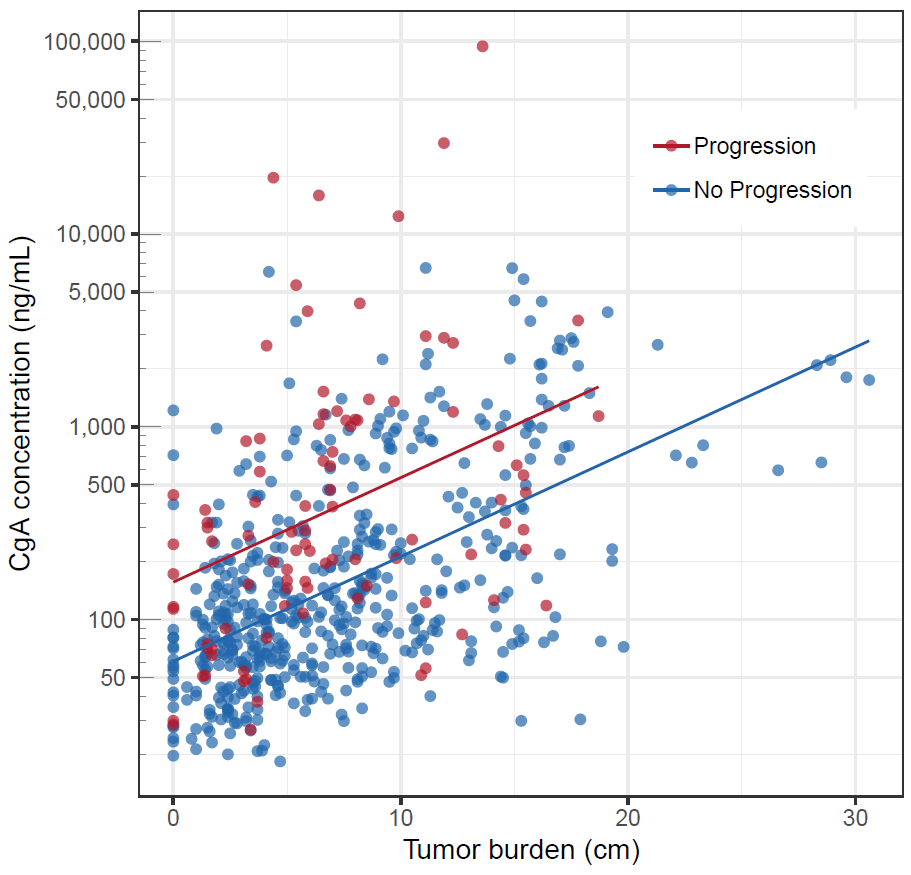


Scatter plot showing the correlation between serum CgA concentration (ng/mL; log-scaled axis) and tumor burden (sum of longest diameter of lesions per RECIST 1.1., in cm) for baseline and follow-up visits of the intention-to-diagnose population. Color coding according to disease state. Patient stratum without progression (blue dots) includes baseline visits. Lines show the fitted linear regression in patient strata.

**Figure S2**


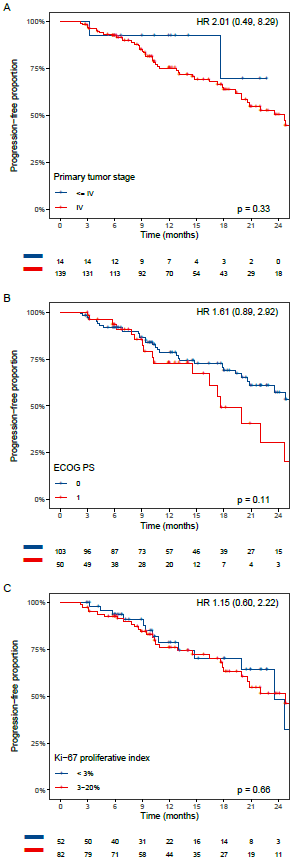


Additional Kaplan-Meier plots for stratification by primary tumor stage (A), ECOG (B) and Ki-67 proliferative index (C). ECOG, Eastern Cooperative Oncology Group.

| Cancer type(s)/subtype(s)/stage(s)/condition | Gastroenteropancreatic neuroendocrine tumors (GEP-NETs) |
| --- | --- |
| Considerations related to: | |
| Sex | Female patients appear to be more prone to developing NETs of the stomach, appendix, or cecum, whereas NETs of the jejunum/ileum, duodenum, and rectum prevail in male patients. |
| Age | GEP-NETs represent the second most common digestive cancer in terms of prevalence. In a series of 64,971 NETs reported to the Surveillance, Epidemiology, and End Results (SEER) program of the National Cancer Institute, the reported annual age-adjusted incidence rate grew from 1.09 per 100,000 in 1973 to 6.98 per 100,000 in 2012. |
| Race/ethnicity | Whereas midgut NETs occur predominantly in white patients, rectal NETs develop more frequently in African American, Asian, and Native American patients. |
| Geography | Epidemiological inconsistencies have been reported between American and European countries versus Asian countries, where a higher incidence of rectal primaries is registered. |
| Other considerations: | The European Neuroendocrine Tumor Society considers CgA to be the most practical and useful general serum tumor marker in patients with NET. We searched PubMed on May 17th, 2023, without date or language restrictions, using the search terms “neuroendocrine tumors” AND “chromogranin A” AND “follow-up” AND “biomarker” to identify reports assessing the utility of serum CgA during follow-up of patients with GEP-NET. 219 publications were identified, only six of which report pertinent CgA data.  Most of the studies identified were monocentric, retrospective, and included small, heterogeneous patient populations. If studies lacked overall methodological quality, they were excluded. As a result, data currently available regarding the usefulness of circulating CgA during follow-up is limited and inconclusive. Expert consensus guidelines have offered varying advice on the use of CgA to practicing clinicians. Therefore, the aim of the CASPAR study was to validate the performance of an automated CgA immunofluorescence assay (B·R·A·H·M·S CgA II KRYPTOR) to monitor the disease course in patients with primary, well-differentiated GEP-NETs. This is the first prospective, multi-center validation of CgA for a clinical algorithm in monitoring the progression of patients with advanced GEP-NET. |
| Overall representativeness of this study | The age distribution of our study (median age of 63) is similar to the average age distribution of GEP-NETs in the literature. |
| **Table S3_ Representativeness of Study Participants** | |
